# Validity of outcome measures used in randomized clinical trials and observational studies in degenerative lumbar spinal stenosis

**DOI:** 10.1038/s41598-022-27218-3

**Published:** 2023-01-19

**Authors:** M. M. Wertli, D. Rossi, J. M. Burgstaller, U Held, N. H. Ulrich, M. Farshad, J. Steurer, F. Brunner

**Affiliations:** 1 Division of General Internal Medicine, Department of General Internal Medicine, Bern University Hospital, Bern University, 3010 Bern, Switzerland; 2 Horten Centre, University of Zurich, Zurich, Switzerland; 3 Department of Internal Medicine, Kantonsspital Baden, Baden, Switzerland; 4 MedX Notfallpraxis, Zurich, Switzerland; 5 Institute of Primary Care, University of Zurich and University Hospital Zurich, Zurich, Switzerland; 6 Department of Biostatistics at Epidemiology, Biostatistics and Prevention Institute, University of Zurich, Zurich, Switzerland; 7 Department of Orthopaedics, Balgrist University Hospital, University of Zurich, Zurich, Switzerland; 8 Department of Physical Medicine and Rheumatology, Balgrist University Hospital, University of Zurich, Zurich, Switzerland

**Keywords:** Medical research, Rheumatology

## Abstract

It is unclear whether outcome measures used in degenerative lumbar spinal stenosis (DLSS) have been validated for this condition. Cross-sectional analysis of studies for DLSS included in systematic reviews (SA) and meta-analyses (MA) indexed in the Cochrane Library. We extracted all outcome measures for pain and disability. We assessed whether the studies provided external references for the validity of the outcome measures and the quality of the validation studies. Out of 20 SA/MA, 95 primary studies used 242 outcome measures for pain and/or disability. Most commonly used were the VAS (n = 69), the Oswestry Disability Index (n = 53) and the Zurich Claudication Questionnaire (n = 22). Although validation references were provided in 45 (47.3%) primary studies, only 14 validation studies for 9 measures (disability n = 7, pain and disability combined n = 2) were specifically validated in a DLSS population. The quality of the validation studies was mainly poor. The Zurich Claudication Questionnaire was the only disease specific tool with adequate validation for assessing treatment response in DLSS. To compare results from clinical studies, outcome measures need to be validated in a disease specific population. The quality of validation studies need to be improved and the validity in studies adequately cited.

## Introduction

Degenerative lumbar spinal stenosis (DLSS) is defined by diminished space for the neural and vascular elements in the central canal of the lumbar spine secondary to degenerative changes of the facet joints, ligaments, vertebrae, and intervertebral discs^1,2^. DLSS is a common disease in elderly patients and typically presents with neurogenic claudication symptoms including pain in the buttocks and lower extremities provoked by walking or extended standing and relieved by rest and bending forward^3^. The treatment options range from nonsurgical approaches such as analgesics, physiotherapy, and epidural corticosteroid injections to surgical methods.

In the past, a multitude of studies assessed the effects of these treatment options for DLSS. In order to be able to establish firm and stringent evidence-based clinical guidelines on the cost-effective use of treatment interventions, results based on clinical trials need to be compared. This is particularly important in systematic reviews and meta-analyses where conclusions are based on the available studies^4^. However, many trials use different outcome measures which complicate the comparison of trial results. Further, studies may use measures that were not validated in the DLSS population and therefore, may not identify clinically relevant changes or differences in this patient population. Indeed, one study showed that depending on the outcome measure that was used and the cut-off values for clinically important improvement, the conclusion of a study may be strongly influenced^5^. To date, no study has systematically assessed the outcome measures used in clinical studies for DLSS and their validation specifically for DLSS.

We performed a cross-sectional analysis of treatment studies for DLSS included in systematic reviews and meta-analyses published between 2006 and April 2021. After extracting the outcome measures for the domains of pain and disability, we assessed whether these instruments were validated specifically for DLSS and critically appraised the quality of the validation studies.

## Methods

### Study design and eligibility criteria

Cross-sectional analysis of outcome measures for pain and disability in treatment studies for DLSS. We included randomized controlled studies (RCT) and observational studies (OS) which were previously included in systematic reviews (SR) or meta-analyses (MA) and were published in the Cochrane library. This approach allowed us to include a complete set of studies for each treatment intervention that was previously assessed for their methodological validity. Spinal stenosis caused by other conditions than degenerative origin (e.g. traumatic, congenital, spondylolisthesis) and other study designs were excluded. This study is not a systematic review, however, reporting will be based, if applicable, on the recommendations of the Preferred Reporting Items for Systematic reviews and Meta-Analysis Protocols (PRISMA statement)^6^ and the Statement for Strengthening the Reporting of Observational Studies in Epidemiology (STROBE statement)^7^.

### Search strategy

We searched for SR and MA assessing surgical and non-surgical treatments for DLSS published in the Cochrane library from its inception (1996) to April 2021. An update search which did not identify additional SR or MA was conducted on June 21, 2022.

Search terms included “lumbar spinal stenosis” in the title, abstract, or keywords and MeSH term “spinal stenosis”.

### Selection process

Two reviewers (DR and MW) independently screened the titles and abstracts for eligible SR and MA according to the pre-defined inclusion criteria. Subsequently, two reviewers (DR and FB) extracted all RCT and OS from the included SR respectively MA into an Endnote database for the analysis. The full text of all RCT and OS were then reviewed for inclusion by DR and confirmed by FB. In case of inconclusive or uncertain eligibility or discrepancies, studies were discussed between the two reviewers and resolved by consensus or by a third party (MW).

If necessary, authors of protocols for systematic reviews and meta-analysis were contacted for further information.

### Data extraction process

The following information was systematically extracted by one reviewer (DR): Author, publication year, study design, treatment intervention, outcome measures for pain and disability, references for validation studies. A second reviewer confirmed the extracted information (FB). Subsequently, all cited validation studies were retrieved and read in full text.

### Quality of validation study

Two reviewers (DR and MW) analyzed the methodological quality of the validation process using the COnsensus-based Standards for the selection of health status Measurement Instruments (COSMIN, https://www.cosmin.nl/tools/checklists-assessing-methodological-study-qualities, assessed on December 2, 2022) checklist^8^. The COSMIN checklist was developed to assess the methodological quality of studies on measurement properties of health-related patient reported outcomes. We extracted information on eight domains: the content validity, internal consistency, construct validity, criterion validity, reliability, responsiveness, flooring/ceiling effect, and interpretability.

*Content validity* Was there a clear description of the measurement aim, the target population, widely accepted or appropriate methods and concepts were used, the item selection, and the investigators / experts involved in item selection are reported. Number of patients adequate (very good ≥ 50, adequate 30–49).

*Internal consistency* Scale or subscale is unidimensional. Were factor analyses performed in an adequate sample (≥ 100 patients very good, adequate 50–99) and Cronbach’s alpha(s) calculated per dimension (Cronbach’s alpha(s) 0.70–0.95)?

*Criterion validity* Was a correlation with the gold standard assessed (at least ≥ 0.70)? Number of patients adequate (≥ 50 very good, 30–49 adequate).

*Construct validity* Were pre-specified hypotheses defined and the results in ≥ 75% in correspondence with these hypotheses (target sample size for this (sub)group analysis ≥ 50 patients)?

*Reliability* Two independent measurements in similar conditions. Was a test–retest intraclass correlation coefficients (ICC)) or weighted Kappa calculated (at least ≥ 0.70, sample size ≥ 50 patients)?

*Responsiveness* Proposed criterion can be considered as a reasonable gold standard. Was the ability to detect a clinical important change over time assessed (AUC ≥ 0.70 or Gyatt’s responsiveness ratio > 1.96)? Number of patients adequate (very good ≥ 50, adequate 30–49)?

Floor or ceiling effects: Was a floor or ceiling effect assessed and not detected (sample size ≥ 50 patients)?

*Interpretability* Was the degree to which one can assign qualitative meaning to quantitative scores assessed (anchor-based method recommended, to determine the minimal clinical difference; sample size ≥ 50 patients)?

Two reviewers (DR and MW) independently assessed each domain and rated the domain as fulfilled (+ , defined as very good or adequately addressed), not fulfilled (-, doubtful or inadequate), not applicable (NA), and nor reported (NR). Disagreement between the reviewers were discussed and resolved by consensus. In case no consensus could be reached, the study was discussed with a third reviewer (FB). All disagreements were resolved by consensus. Finally, a quality score was calculated ranging from 0 (no domain was fulfilled) to 8 points (all domains were fulfilled).

### Outcome of interest

Primary outcome were outcome measures in the domains of pain and disability.

### Data synthesis

We summarized categorical variables with number and percentage and continuous data with mean and standard deviation. All analyses were conducted with the statistical software R (version 3.6.1).

## Results

### Study selection

The literature search in the Cochrane library retrieved 31 eligible references. Twenty references met our inclusion criteria and were included in the study (systematic reviews n = 15, meta-analysis n = 3, combined systematic review and meta-analysis n = 2). Subsequently, a total of 256 primary studies were extracted for full-text assessment. For details see Table 1.

**Table 1:** Characteristics of included SR and/or MA (n = 20).

	References	SR/MA	Number of included studies
1	Ammendolia et al.^62^	SR	21
2	Ammendolia et al.^63^	SR	18
3	Chou et al.^64^	SR	5
4	Helm et al.^65^	SR	7
5	Hong et al.^66^	MA	21
6	Iversen et al.^67^	SR	6
7	Jarrett et al.^68^	SR	13
8	Kim et al.^69^	SR/MA	12
9	Kovacs et al.^70^	SR	5
10	Kreiner et al.^71^	SR	13
11	Macedo et al.^72^	SR	10
12	Machado et al.^73^	SR	24
13	May and Comer^74^	SR	31
14	McGregor et al.^75^	SR	3
15	Moojen et al.^76^	SR/MA	11
16	Overdevest et al.^77^	SR	10
17	Podichetty et al.^78^	MA	4
18	Reiman et al.^79^	SR	11
19	Wu et al.^80^	MA	5
20	Zaina et al.^81^	SR	26
Total			256

*SR* systematic review; *MA *meta-analysis.

**Figure 1:**
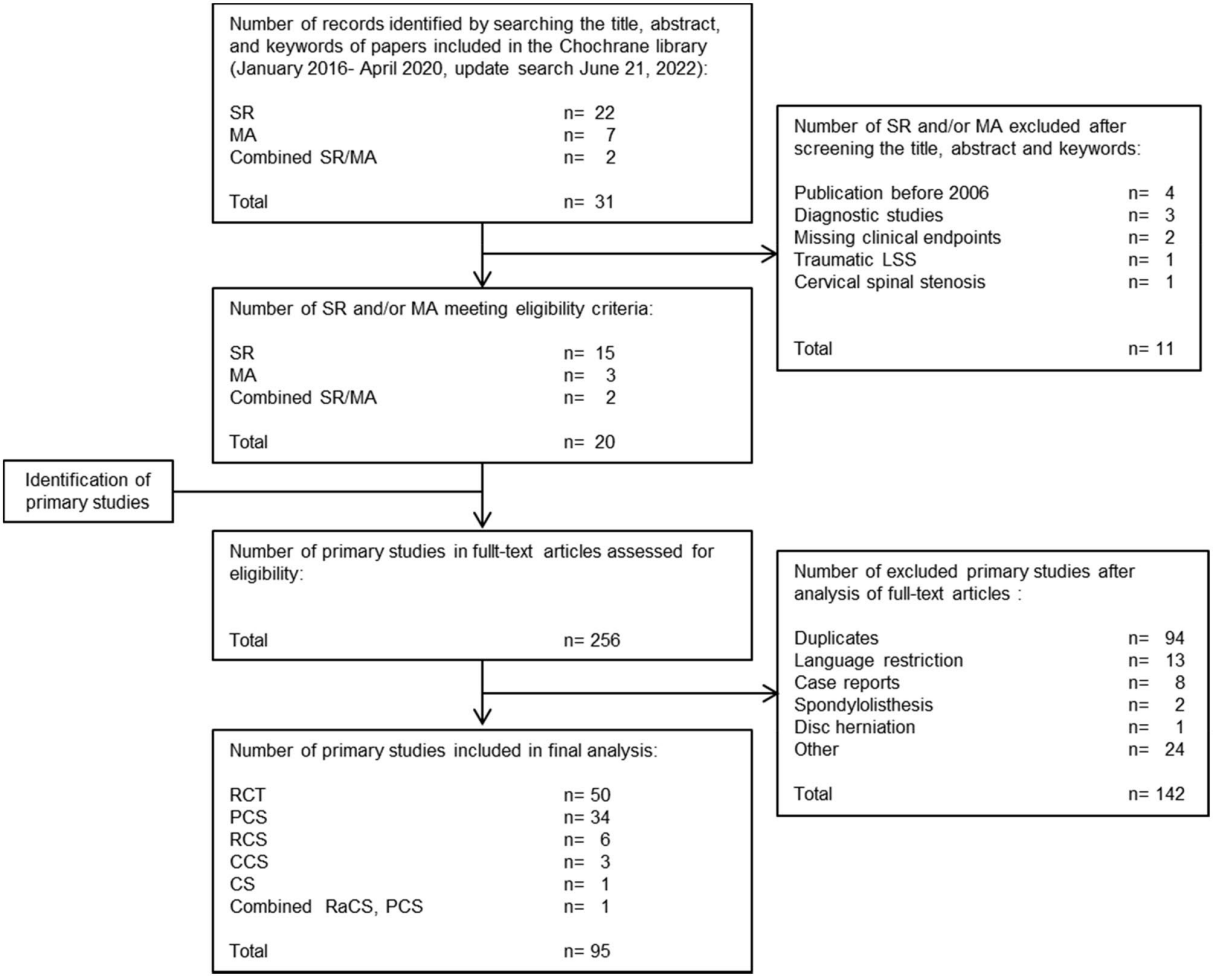
Flow chart.

After full text screening, 95 primary studies were included in the final analysis. One hundred and forty-two studies did not fulfill our inclusion criteria and were excluded. The main reason for exclusion were duplicates (n = 94). The study selection process is depicted in Fig. 1.

### Characteristics of the included primary studies

The characteristics of the included primary studies are summarized in Table 2. Most of the studies were randomized controlled trials (n = 50, 48.5%) and prospective cohort studies (n = 34, 35.8%). Almost three quarters (73%) of the primary studies involved at least one surgical intervention. Studies were published between 1983 and 2016.

**Table 2:** Characteristics of the included studies.

References	Study design	Intervention/control group	Number of participants	Age (years)	Follow-up (weeks)	Outcome measure
Forsth et al.^82^	RCT	Decompression, fusion/decompression	247	66.9	96	VAS, ZCQ, walking tolerance
Komp et al.^83^	RCT	Decompression: full-endoscopic interlaminar technique/conventional microsurgical laminotomy technique	135	62	96	VAS, NASS, ODI
Lonne et al.^84^	RCT	Minimally invasive decompression/X-Stop	96	67	96	NRS, ODI
Mobbs et al.^85^	RCT	Conventional laminectomy/microscopic unilateral laminectomy	79	69.3	96	VAS, ODI
Richter et al.^12^	RCT	Decompressive surgery/decompressive surgery with interspinous device	62	68	96	VAS, ODI, RMQ, walking tolerance
Beyer et al.^14^	PCS	Open decompression/percutaneous interspinous spacer	45	69.3	96	VAS, ODI, SF-36, walking tolerance
Chopko^86^	PCS	Percutaneous lumbar decompression	45	70.1	96	VAS, ODI, ZCQ
Davis et al.^87^	RCT	Laminectomy interlaminar stabilization (Coflex)/laminectomy with posterior spinal fusion	322	63	96	VAS, ODI, ZCQ, SF-12
Durkin et al.^88^	RCS	Minimally invasive lumbar decompression (MILD)	50	73.3	24	NRS, PROMIS, ODI, ZCQ
Liu et al.^89^	RCT	Modified unilateral laminotomy for bilateral decompression	56	60	96	VAS, JOABPEQ
Moojen et al.^38^	RCT	Interspinous device implantation/conventional decompression	159	67.5	52	VAS, MGPQ, ZCQ, SF-36, RMQ, walking tolerance
Rajasekaran et al.^90^	RCT	Lumbar spinous process splitting decompression (LSPSD)/conventional midline decompression	51	56	56.8	VAS, JOABPEQ, NCOS
Stromqvist et al.^91^	RCT	Indirect compression (X-Stop)/conventional decompression	100	69	96	VAS, ODI, ZCQ, SF-36
Wang et al.^92^	RCS	Minimal invasive lumbar decompression (MILD)	22	74.2	38.2	VAS
Basu^93^	PCS	Minimal invasive lumbar decompression (MILD)	27	63.3	24	VAS, ODI, ZCQ
Brown^94^	RCT	Epidural steroid injection/minimal invasive lumbar decompression (MILD)	38	76.2	12	VAS, ODI, ZCQ
Deer et al.^95^	PCS	Minimal invasive lumbar decompression (MILD)	46	66.1	48	VAS, ODI, ZCQ
Gurelik et al.^21^	RCT	Unilateral laminotomy/decopressive laminectomy	52	59	36.4	ODI, walking tolerance
Kim et al.^96^	PCS	Spinal fusion with interspinous fusion device, posterior lumbar interbody fusion (PLIF)/spinal fusion with pedicle screw fixation	76	55.8	64.7	VAS, ODI
Mekhail et al.^39^	PCS	Percutaneous decompression	58	70.0	48	VAS, ODI, ZCQ, SF-12
Mekhail et al.^97^	PCS	Percutaneous decompression	40	72.2	40	PDI, RMQ, VAS, standing time, walking tolerance
Wilkinson and Fourney^98^	PCS	Percutaneous remodeling of ligamentum flavum and lamina	10	64	26	VAS, ODI, SF-12
Wong,^99^	CS	Minimally invasive lumbar decompression (MILD)	17	73.1	48	VAS, ODI
Aalto et al.^100^	PCS	Rehabilitation group/standard postoperative treatment	102	62.5	96	NRS, ODI
Chopko^101^	PCS	Percutaneous remodeling of ligamentum flavum and lamina	14	69	23.5	VAS, ODI
Holinka et al.^22^	PCS	Dynamic interspinous spacers, interlaminar decompression/interlaminar decompression	50	72	180	VAS, ODI, walking tolerance
McGregor et al.^102^	RCT	Usual care/booklet /rehabilitation/booklet, rehabilitation	338	53.8	52	ODI, VAS
Postacchini et al.^103^	RCT	Aperius interspinous implant/open decompression	71	67	104	ODI, ZCQ
Slatis et al.^31^	RCT	Laminectomy, transpedicular-instrumented fusion/non-operative	94	62.5	288	VAS, ODI, NSR, walking tolerance
Watanabe et al.^104^	RCT	Split laminectomy /conventional laminectomy	41	70	1	VAS, JOABPEQ
Azzazi and Elhawary^105^	RCT	Dynamic stabilization (X-Stop)/transpedicular screw fixation	60	56.3	96	VAS, ODI
Celik et al.^15^	CCS	Bilateral microdecompressive laminotomy/laminectomy	71	60	256.9	VAS, ODI, walking tolerance
Chopko and Caraway^106^	PCS	Minimal invasive lumbar decompression (MILD)	78	70	6	VAS, ODI, ZCQ, SF-12
Comer et al.^16^	RCT	Walking stick/control	46	71.26	60	VAS, ODI, ZCQ, walking tolerance
Galarza et al.^107^	PCS	Decompression (Aperius PercLID System)	40	72.7	64	VAS, ZCQ
Goren et al.^19^	RCT	Exercise/exercise, ultrasound	45	53.2	3	VAS, ODI, walking tolerance
Lingreen and Grider^40^	RCS	Minimal invasive lumbar decompression (MILD)	42	52–86	2	VAS, walking tolerance, standing time
Richter et al.^29^	CCS	Decompressive surgery/decompressive surgery, interspinous device (Coflex)	60	68	48	VAS, ODI, RMQ, walking tolerance
Ryu and Kim^108^	PCS	One level unilateral laminotomy bilateral decompression/one level unilateral laminotomy bilateral decompression, device for intervertebral assisted motion	36	70.57	88.7	VAS
Sobottke et al.^33^	PCS	Open microsurgical decompression/implantation of interspinous stand-alone spacer	36	68.1	48	VAS, ODI, SF-36, walking tolerance
Weinstein et al.^109^	Ra,CS, PCS	Decompressive laminectomy, non-operative care	654	65.5	192	ODI, SF-36
Koc et al.^23^	RCT	Physical therapy/epidural steroid injection/control	29	59.1	24	VAS, RMQ, FFD, STS, WCT, walking tolerance
Kuchta et al.^110^	RCS	Interspinous spacer implantation (X-Stop)	175	69.4	96	VAS, ODI
Lee et al.^111^	RCT	Epidural steroid injections: translaminar, caudal, transforaminal	192	52.54	16	NRS, R5PS
Levendoglu^24^	PCS	Lumbar corset	70	59.23	NR	Walking tolerance
Manchikanti et al.^112^	RCT	Percutaneous epidural adhesiolysis/fluoroscopically directed caudal epidural injections	50	52	48	NRS, ODI
Manchikanti et al.^113^	RCT	Epidural injection (local anesthetic, steroids, 0.9% sodium chloride)/percutaneous adhesiolysis with lidocaine, 10% hypertonic sodium chloride, betamethasone)	120	61.5	48	NRS, ODI
Matsudaira^114^	RCT	Limaprost/Etodolac	79	59.2	8	SF-36
Park et al.^115^	RCS	Posterior dynamic stabilization/posterior lumbar interbody fusion	61	63	157.5	VAS, ODI
Sahin,^30^	RCT	Physical therapy/ physical therapy, calcitonin	45	56.1	8	VAS, RMQ, walking tolerance
Tafazal et al.^10^	RCT	Periradicular injection: Bupivacaine, methylprednisolone/bupivacaine	124	51.9	80	VAS, LBOS, ODI
Yagi et al.^116^	PCS	Modified unilateral midline decompression	41	72	72.8	VAS, JOABPEQ
Yasar et al.^37^	PCS	Decompressive surgery	125	58	48	VAS, ODI, walking tolerance
Bhadra et al.^117^	PCS	Interspinous process distraction (X-Stop)	45	61.5	48	VAS, ODI, SF-12
Brussee et al.^118^	PCS	Interspinous process distraction (X-Stop)	65	64.4	48	ZCQ, SF-36
Fu et al.^41^	PCS	Laminoforaminotomy/decompressive surgery	152	57	160	VAS, ODI, walking tolerance
Yano et al.^119^	PCS	Ceramic interspinous process spacer	19	70.1	149.6	VAS, ZCQ
Athiviraham and Yen^120^	PCS	Decmpression/decompression, fusion/ conservative	112	67	96	RMQ
Cavusoglu et al.^121^	PCS	Bilateral decompression	50	69.81	91.2	VAS, ODI, SF-36
Cho et al.^122^	RCT	Split-spinous process laminotomy, discectomy/conventional laminectomy with or without discectomy	70	60.2	59.9	VAS, JOABPEQ
Kim et al.^123^	CCS	Laminectomy and/or microdiscectomy/dynamic interspinous spacer, laminectomy and/or microdiscectomy	62	50	48	VAS
Kong et al.^124^	RCT	Interspinous implant (Coflex)/posterior lumbar interbody fusion	42	58	48	VAS, ODI
Malmivaara et al.^9^	RCT	Decompression/nonoperative treatment	94	62.5	96	VAS, ODI, walking tolerance
Mannion et al.^125^	RCT	Postoperative rehabilitation: spine stabilization exercises/mixed techniques/self-management	165	60.8	96	VAS, RMQ
Pua et al.^126^	RCT	Treadmill with body weight support/cycling	68	58.4	6	VAS, ODI, RMQ
Siddiqui et al.^127^	PCS	Interspinous implant (X-Stop)	40	71.5	48	ODI, ZCQ, SF-36
Tafazal et al.^11^	RCT	Nasal salmon calcitonin/placebo	40	68.6	16	VAS, LBOS, ODI, walking tolerance
Yaksi et al.^36^	RCT	Gabapentin and standard treatment/standard treatment	55	50.8	16	VAS, walking tolerance
Anderson et al.^128^	RCT	X-Stop/nonoperative	75	69.2	96	ZCQ, SF-36
Hsu et al.^129^	RCT	X-Stop/nonoperative	191	70	96	SF-36
Kondrashov et al.^130^	RCS	X-Stop	18	67	204	ODI, ZCQ, SF-36
Murphy et al.^131^	PCS	Distraction mobliziation, neural mobilization	55	65.2	66	NRS, RMQ
Veihelmann et al.^132^	RCT	Epidural neuroplasty/physiotherapy	99	44	48	VAS, ODI
Whitman et al.^35^	RCT	Manual physical therapy, body weight supported treadmill walking, exercise/lumbar flexion exercises, treadmill walking program, subtherapeutic ultrasound	58	69.5	48	NRS, ODI, ZCQ, walking tolerance
Atlas et al.^133^	PCS	Surgery/nonoperative	97	65.6	480	SF-36, RMQ, SBS
Gerdesmeyer et al.^134^	PCS	Percutaneous minimally invasive neurolysis	61	49	24	ODI
Ng et al.^25^	RCT	Periradicular Infiltration: bupivacaine, methylprednisolone/ bupivacaine	86	50.45	12	VAS, ODI, walking tolerance
Paker et al.^26^	RCT	Surgery (decompression, laminectomy)/nonoperative	41	66.19	113.5	VAS, walking tolerance
Thome et al.^135^	RCT	Bilateral laminotomie /unilateral laminotomie/laminectomie	120	68	62	VAS, SF-36, RMQ
Zucherman et al.^136^	RCT	X- Stop/nonoperative	191	69.3	48	ZCQ, SF-36
Lee et al.^137^	PCS	X-Stop	10	71	44	ZCQ
Manchikanti et al.^138^	RCT	Catheterization without adhesiolysis, injection: local anesthetics, normal saline, steroid/catheterization with adhesiolysis, injection: local anesthetics, normal saline, steroid/adhesiolysis, injection: local anesthetic, hypertonic saline, steroid	75	47	48	VAS, ODI
Podichetty et al.^27^	RCT	Calcitonin/placebo	55	68.7	12	VAS, ODI, SF-36, walking tolerance
Mariconda et al.^139^	RCT	Unilateral laminectomy/nonoperative	44	61	192	BSS
Prateepavanich et al.^28^	PCS	Corset/no corsett	21	62.5	1	VAS, walking tolerance
Amundsen et al.^13^	PCS	Operative/nonoperative	100	59	480	VAS, walking tolerance
Simotas et al.^140^	PCS	Nonoperative	49	69	132	VAS, RMQ
Waikakul et al.^34^	RCT	Methylcobalamin/Kontrolle	152	67	96	Walking tolerance
Heavner et al.^141^	RCT	Percutaneous epidural neuroplasty: NaCl 0.9%/NaCL 10%/with and without hyaluronidase	59	54	48	VAS, MGPQ
Fukusaki et al.^18^	RCT	Epidural injection: NaCl/mepicacaine/mepivacaine, methylprednisolone	53	70.3	12	Walking tolerance
Amundsen et al.^142^	RCT	Plain radiography/myelography/computed tomographic imaging	100	NR	NR	VAS
Grob et al.^20^	RCT	Decompression/decompression with arthrodesis most stenotic segment/ decompression of all segments	45	67	112	VAS, walking tolerance
Eskola et al.^17^	RCT	Calcitonin subcutaenous/NaCl subcutaneous	39	56.6	48	VAS, walking tolerance, DECT
Porter and Miller^143^	RCT	Calcitonin subcutaneous/NaCl subcutaneous	42	55.2	8	VAS, walking tolerance
Porter & Hibbert 1983^144^	PCS	Calcitonin	41	55	10	VAS, ODI, walking tolerance

*RCT* randomized controlled study; *PCS *prospective cohort study; *RCS *retrospective cohort study; *CCS *case control study; *CS *case series; *RaCS *randomized cohort study; *NR *not reported; *BSS* Beaujon scoring system; *DECT* Digitest ergojump contact test; *FFD* Finger floor distance; *JOA* Japanese orthopedic association back pain evaluation questionnaire; *LBOS* Low back outcome score; *MGPQ* McGill pain questionnaire; *NASS* North American spine society instrument; *NCOS* Neurgenic claudication outcome score; *NRS* Numeric rating scale; *ODI* Oswestry disability index; *PDI* Pain disability index; *PROMIS* Patient reported outcomes measurements information s ystem; *RMQ* Roland Morris questionn aire; *R5PS* Roland 5-point pain score; *SF-12* Short form-12; *SF-36* Short fo rm-36; *VAS* Visual analogue scale; *ZCQ* Zurich claudication questionnaire.

The primary studies included a total of 7′878 participants with a median age of 63.5 ± 7.1 years (range 44–76.2 years). The median follow-up duration was 78.1 ± 81.3 weeks (range 1–480 weeks).

Table 3 summarizes the outcome measures used in the primary studies. In total, 242 outcome measures were identified. In the domain of pain four outcome measures were detected. The Visual Analogue Scale (VAS, n = 69, 90%) respectively Numeric Rating Scale (NSR, n = 9, 9%) were most commonly used. In the domain of disability, a total of 12 outcome parameters were identified. The Oswestry Disability Index (ODI, n = 53, 47%) and various tests assessing walking tolerance (n = 34, 29%) were mostly used (walking ability^9–11^, pain free walking^12^, walking distance^13–37^, walking test^38^, walking time^39^, walking < 15 minutes^40^, walking tolerance ^41^).

**Table 3:** Outcome measures in the domain of pain and disability.

Domain	Outcome	Number of uses in primary studies	Reference of primary studies in which a reference was cited for an outcome	Reference for outcome	Reference for DLSS specific validation study
Pain (n = 4)	VAS	69	^31,102,103,127,132^	^145–148^	
	NRS	9	^112,113^	^149–154^	
	MGPQ	2			
	R5PS	1			
Disability (n = 12)	ODI	53	^9–11,19,21,27,31,35,39,82–85,100,102,109,112,113,126,127,134,144^	^43,46,155–164^	^43,46,47^
	Walking tolerance	34	^23,82^	^47,165,166^	^43,163,164,167^
	RMQ	13	^23,97,120,125,126,131,133,140^	^168–173^	^45^
	JOABPEQ	5			
	Standing time	2			
	WCT	1	^23^	^164^	^164^
	NCOS	1			
	STS	1	^23^	^164^	^164^
	DECT	1			
	FFD	1			
	PDI	1	^97^	^173,174^	
	PROMIS	1			
Pain and disability (n = 6)	ZCQ	22	^35,38,82,84,91,93–95,106,118,119,128,136,137^	^42–45^	^42–45^
	SF-36	16	^27,109,114,118,129^	^175–182^	
	SF-12	5			
	LBOS	2	^10^	^183,184^	
	BSS	1	^139^	^167^	^167^
	NASS	1	^83^	^185,186^	
Total	23	242	64	58	14

*BSS* Beaujon scoring system; *DECT* Digitest ergojump contact test; *FFD* Finger floor distance; *JOA* Japanese orthopedic association back pain evaluation questionnaire; *LBOS* Low back outcome score; *MGPQ* McGill pain questionnaire; *NASS* North American spine society instrument; *NCOS* Nlaudicationaudicatio outcome score; *NRS* Numeric rating scale; *ODI* Oswestry disability index; *PDI* Pain disability index; *PROMIS* Patient reported outcomes measurements information s ystem; *RMQ* Roland Morris questionn aire; *R5PS* Roland 5-point pain score; *SF-12* Short form-12; *SF-36* Short fo rm-36; *VAS* Visual analogue scale; *ZCQ* Zurich claudication questionnaire.

In the domain of pain and disability combined, the Zurich Claudication Questionnaire (ZCQ, n = 22, 47%) and the SF-36 (n = 15, 32%) were frequently applied.

### Outcome measures and reference studies

In total, 45 primary studies (47.3%) provided a reference for at least one outcome measure. In the domain of pain references were provided for the VAS (n = 5) and the NRS (n = 2), respectively. In the domain of disability, the ODI (n = 22) and the Roland Morris Disability Questionnaire (RMQ, n = 8) were most frequently referenced. In the domain of pain and disability combined the ZCQ (n = 14) was commonly referenced.

For nine outcome measures (disability n = 7, pain and disability combined n = 2) a total of 14 validation studies specifically for a DLSS population were found. For the ZCQ (n = 4)^42–45^ and the ODI (n = 3)^43,46,47^ more than one validation study was identified. For details see Table 4.

**Table 4:** Summary and quality of validation studies.

Outcome measure	ODI			RMQ	TWT	STS	WCT	SPWT	SWT	ZCQ				BSS
Publication (Author, year, Reference)	Pratt et al.^43^	Fritz et al.^47^	Fairbanks et al.^46^	Stucki et al.^45^	Whitehurst et al.^164^	Whitehurst et al.^164^	Whitehurst et al.^164^	Tomkins et al.^163^	Pratt et al.^43^	Stucki et al.^44^	Stucki et al. ^45^	Pratt et al.^43^	Comer et al.^42^	Lassale et al.^167^
Number of participants	52	45	550	193	123	123	123	33	52	193	193	52	99	314
Content validity^1^	** + **	** + **	NR	** + **	** + **	** + **	** + **	** + **	NR	** + **	** + **	** + **	** + **	** + **
Internal consistency^2^	** + **	−	** + **	NR	NR	NR	NR	−	** + **	** + **	** + **	** + **	** + **	NR
Criterion validity^3^	NR	NR	NR	NR	NR	NR	NR	** + **	** + **	NR	NR	NR	NR	NR
Construct validity^4^	NR	−	NR	NR	NR	NR	NR	−	NR	** + **	** + **	NR	NR	NR
Reliability^5^	** + **	−	NR	NR	** + **	** + **	** + **	** − **	** + **	** + **	** + **	** + **	NR	NR
Responsiveness^6^	NR	** + **	NR	** + **	NR	NR	NR	−	NR	+	+	NR	NR	** + **
Floor or ceiling effects^7^	NR	NR	NR	** + **	NR	NR	NR	NR	NR	NR	** + **	NR	NR	NR
Interpretability^8^	NR	−	NR	NR	NR	NR	NR	−	NR	** + **	** + **	NR	NR	NR
**Quality score (0/8–8/8)**	3/8	2/8	1/8	3/8	2/8	2/8	2/8	2/8	3/8	6/8	7/8	3/8	2/8	2/8

**Interpretation (COSMIN Checklist)**^8^**.**

+ , domain fulfilled (very good or adequately addressed); NA, not applicable; NR, not reported; − domain was not fulfilled.

^1^Content validity: clear description of the measurement aim, the target population, widely accepted or appropriate methods and concepts were used, the item selection, and the investigators OR experts involved in item selection. Number of patients adequate (very good ≥ 50, adequate 30–49).

^2^Internal consistency: Scale or subscale was unidimensional. Factor analyses performed on adequate sample size (≥ 100 patients very good, adequate 50–99) AND Cronbach’s alpha(s) calculated per dimension AND Cronbach’s alpha(s) between 0.70 and 0.95.

^3^Criterion validity: Correlation with the gold standard is at least 0.70? Number of patients adequate (≥ 50 very good, 30–49 adequate)?

^4^Construct validity: hypotheses are pre-specified; ≥ 75% of the results are in correspondence with these hypotheses, in (sub)groups of ≥ 50 patients.

^5^Reliability: Two independent measurements in similar conditions. Test–retest intraclass correlation coefficients (ICC)) or weighted Kappa is at least 0.70 in a sample size ≥ 50 patients.

^6^Responsiveness: Proposed criterion can be considered as a reasonable gold standard. Was the ability to detect a clinical important change over time assessed (AUC ≥ 0.70 or Gyatt’s responsiveness ratio > 1.96)? Number of patients adequate (very good ≥ 50, adequate 30–49)?

^7^Floor or ceiling effects: absence of floor and ceiling effects if no floor or ceiling effects are present in ≥ 50 patients.

^8^Interpretability: Degree to which one can assign qualitative meaning to quantitative scores (anchor-based method recommended, to determine the minimal clinical difference). Sample size of ≥ 50 patients.

*BSS* Beaujon scoring system; *ODI* Oswestry disability index; *RMQ* Roland Morris questionnaire; *SPWT* Self-paced walking test; *SWT* Shuttle Walking Test; *STS* Sit-to-stand test; *TWT* Treadmill walk test; *WCT* Weight carrying test; *ZCQ* Zurich claudication questionnaire.

### Quality assessment of the validation studies

None of the validation studies assessed all predefined domains of the COSMIN checklist^8^ (Table 4). Twelve of the included 14 studies reached a quality score of 3/8 or less, indicating low methodological quality. None of the validation studies reached the score maximum (range 2/8–7/8). The two studies by Stucki et al.^44,45^ assessing the validation of the ZCQ in DLSS population, achieved the highest scores (6/8 respectively 7/8).

The Beaujon scoring system (BSS) and various tests assessing walking tolerance were tested in a DLSS population. However, the methodology of the validation study was not in agreement with the methodological items proposed for measurements of health-related patient reported outcomes^8^.

## Discussion

### Main findings

The results of this cross-sectional analysis indicate the reporting of outcome measures in randomized clinical trials and observational studies in DLSS is insufficient. Less than half of the included primary studies provided a reference for at least one outcome measure in the domain of pain, disability, or combined pain and disability. A total of 14 validation studies for nine outcome measures were found. The quality assessment of the validation studies revealed low quality for the majority of the studies. Within the DLSS population three validation studies were found for the ODI and four validation studies for the ZCQ, respectively. However, all three validation studies for ODI scored unsatisfactory in the quality assessment. Based on this study, the ZCQ represents the only disease specific tool with adequate validation for assessing treatment response in DLSS.

### Results in light with the literature

The findings of our study are in agreement with a systematic review and meta-analysis on outcome measures for neurogenic claudication^48^. The authors evaluated 15 separate walking outcome measures and concluded that walking outcome measures for patients with neurogenic claudication are lacking. The development of a measurement instrument involves testing validity and reliability with a defined target population^49^. Choosing a measurement instrument wisely can be challenging given the growing number of choices available. Meaningful use of a measurement instrument depends not only on the validity of the instrument itself, but also on the context in which it is used^50^. Web-based systems such as PROMIS have been developed from efforts to optimize and simplify the process of selecting an appropriate measurement instrument^51^. The stated goal is to provide well-constructed, generalizable, and clinically relevant endpoints for studies^52^. These systems facilitate the completion of questionnaires for subjects, as otherwise there would be a considerable administrative burden. In 2006, the North America Spine Society (NASS) Compendium for the Assessment and Research of Spinal Disorders recommended the Quebec Back Pain Disability Scale, the Roland Morris Disability Questionnaire, and the Waddell Nonorganic Signs for lumbar pain as measurement tools^53^. In contrast to lumbar back pain, there are currently no specific recommendations for the use of measurement tools in DLSS^54^. However, measurement tools that are valid for patients with nonspecific back pain do not necessarily measure the relevant endpoints for patients with DLSS. The latter have a different clinical presentation with typical claudication symptoms. Consequently, depending on the conception and design of a questionnaire, clinical outcomes may vary significantly^5^. The variance of measured symptoms can vary widely, as shown in a recently published study^55^. The comparison of measurement instruments in patients with DLSS showed that there was a variability of 40–70% depending on cut-off and measurement instrument. In a recently published study^56^, the ZCQ was the most responsive tool to assess symptoms and function in DLSS supporting the findings of the current systematic analysis. The use of non-validated, nonspecific measurement instruments in studies has an impact on future clinical decisions. The extent of this variation was relevant enough to lead to completely different interpretations of a study. Kimberlin et al.^57^ argue that although any outcome of a measurement instrument is only an approximation of the actual truth, the use of non-validated measurement instruments has the same effect on study quality as a poor study design or an insufficient number of patients. Our study shows that many of the measurement tools used have not been validated in DLSS patients and it is therefore unclear whether they represent what is relevant to patients.

The issue of inclusion of a magnitude of different outcomes in trials of the same intervention is not novel. For example, in their systematic review from 2017 Mayo-Wilson et al.^58^ identified variation in outcomes across reports of RCTs the effect of gabapentin for treating neuropathic pain and quetiapine for bipolar depression, respectively. The authors found that the RCTs included hundreds of outcomes and concluded that researchers may cherry-pick what they report from multiple source of RCT information. This results in challenges for interpreting clinical trials and obstacles in comparing clinical trials in meta-analyses.

The development of a measurement instrument involves testing validity and reliability with a defined target population^49^. Choosing a measurement instrument wisely can be challenging given the growing number of choices available. In recent years, various efforts have been made to systematically assess the validity of measurement instruments^59^. Meaningful use of a measurement instrument depends not only on the validity of the instrument itself, but also on the context in which it is used^50^ Web-based systems such as PROMIS have been developed from efforts to optimize and simplify the process of selecting an appropriate measurement instrument^60^ The stated goal is to provide well-constructed, generalizable, and clinically relevant endpoints for studies.

### Strength and limitations

To the best of our knowledge this is the first cross-sectional analysis of outcome measures used in randomized clinical trials and observational studies in DLSS. In addition, we conducted a validity check of the outcomes applying existing guidelines for conducting systematic literature reviews^51^.

As we focused on systematic reviews and meta-analyses, it is potentially possible that individual studies may not be identified in our analysis. However, we are confident that our methodology included the most relevant papers. The main limitation of this study is that this approach did not capture all validation studies conducted to date. To include an overview of all validation studies ever conducted in patients with DLSS would require a systematic review. By using complete sets of studies included in SR and MA, we assessed the quality of reporting of validation studies and the quality of the validation studies themselves. Therefore, we did not aim to provide a complete overview for all validation studies conducted in DLSS. Thus, when included in this systematic literature review, a study underwent two selection processes.

### Implications for clinical research

In order to assess the effectiveness of treatment studies in patients with DLSS, valid and comparable measurement instruments are central. Our study shows that many different and partly unvalidated instruments are used. In addition, there is a lack of information on the minimal clinically important change of the respective measurement instruments. Researchers should systematically conduct high quality validation studies for the measurement instruments in DLSS patients. In addition, the patients’ perspective should be included in the selection of measurement instruments. Further validation studies of measurement instruments specific for DLSS patients with at least 50 patients and considering the quality criteria of Terwee et al.^61^ will help to quantify the symptoms relevant for DLSS patients and thus have a direct impact on the validity of future RCTs and OS.

### Implications for clinical practice

Increasingly, patient-centered measurement instruments are recommended or required for measuring treatment outcome. Our study shows that the selection of adequately validated measurement instruments for DLSS patients is important and that many measurement instruments are not validated in this patient population. In particular, reliable and valid questionnaires specific to DLSS are helpful for everyday clinical practice, as clinical progress can be monitored and responses are less influenced by the treating individuals. For monitoring treatment response in DLSS, we believe that ZCQ provides the most differentiated results. In particular, this questionnaire has the advantage of combining the assessment of pain, satisfaction and disability at the same time.

## Conclusion

Reporting of the validity of outcome measures was poor and only in validation in one outcome measure was adequate. In order to be able to compare results from clinical studies, outcome measures need to be validated in a disease specific population and external validation studies should be indicated adequately. For monitoring treatment response in DLSS, the use of the ZCQ is recommended.

## Data Availability

The datasets used and/or analyzed during the current study are available from the corresponding author on reasonable request.
